# Supplementary material for: Impact of Serum Lipid on Breast Cancer Recurrence
Source: J Clin Med. 2020 Sep 2;9(9):2846. doi: 10.3390/jcm9092846 (PMC7564113; doi:10.3390/jcm9092846)
Supplement: Supplementary file 1 [file jcm-09-02846-s001.pdf]

Supplementary Table S1. Characteristics by quartile of total cholesterol at baseline in breast cancer patients who underwent a surgery.

	**Overall (*N* = 4,190) N (%)**	**Quartile I (*n* = 1,011) N (%)**	**Quartile II (*n* = 1,074) N (%)**	**Quartile III (*n* = 1,012) N (%)**	**Quartile IV (*n* = 1,093) N (%)**	***p*-Value**
Age, Mean(SD)	51.7 (9.8)	48.9 (10.8)	51.1 (23.4)	52.6 (9.2)	54.0 (8.8)	<0.001
BMI, Mean(SD)	23.6 (3.2)	23.0 (3.2)	23.4 (3.0)	23.7 (3.2)	24.2 (3.2)	<0.001
Menopausal status						<0.001
Yes	2,217 (52.9)	392 (38.8)	522 (48.6)	579 (57.2)	724 (66.2)	
No	1,962 (46.8)	616 (60.9)	550 (51.2)	431 (42.6)	365 (33.4)	
Unknown	11 (0.3)	3 (0.3)	2 (0.2)	2 (0.2)	4 (0.4)	
Comorbidity (n = 2,946)*						0.80
Yes†	636 (21.6)	153 (21.9)	160 (20.7)	168 (22.7)	155 (21.1)	
No	2,310 (78.4)	547 (78.1)	612 (79.3)	572 (77.3)	579 (78.9)	
Surgery type (n = 4,188)						0.68
Mastectomy	1,166 (27.8)	287 (28.4)	298 (27.8)	291 (28.8)	290 (26.5)	
Lumpectomy	3,022 (72.2)	723 (71.6)	775 (72.2)	721 (71.3)	803 (73.5)	
Stage						0.18
1	2,050 (48.9)	505 (50.0)	502 (46.7)	523 (51.7)	520 (47.6)	
2	1,679 (40.1)	384 (38.0)	455 (42.4)	387 (38.2)	453 (41.5)	
3	461 (11.0)	122 (12.1)	117 (10.9)	102 (10.1)	120 (11.0)	
ER						0.01
Positive	3,155 (75.3)	789 (78.0)	825 (76.8)	741 (73.2)	800 (73.2)	
Negative	1,032 (24.6)	221 (21.9)	247 (23.0)	271 (26.8)	293 (26.8)	
Unknown	3 (0.1)	1 (0.1)	2 (0.2)	0 (0.0)	0 (0.0)	
PR						<0.001
Positive	2,894 (69.1)	741 (73.3)	763 (71.0)	681 (67.3)	709 (64.9)	
Negative	1,293 (30.9)	269 (26.6)	309 (28.8)	331 (32.7)	384 (35.1)	
Unknown	3 (0.1)	1 (0.1)	2 (0.2)	0 (0.0)	0 (0.0)	
HER2						0.01
Positive	842 (20.1)	180 (17.8)	199 (18.5)	220 (21.7)	243 (22.2)	
Negative	3,275 (78.2)	812 (80.3)	861 (80.2)	780 (77.1)	822 (75.2)	
Unknown	73 (1.7)	19 (1.9)	14 (1.3)	12 (1.2)	28 (2.6)	
Subtype						<0.001
Luminal A	2,737 (65.3)	690 (68.3)	723 (67.3)	634 (62.7)	690 (63.1)	
Luminal B	406 (9.7)	92 (9.1)	107 (10.0)	106 (10.5)	101 (9.2)	
HER2 type	436 (10.4)	88 (8.7)	92 (8.6)	114 (11.3)	142 (13.0)	
TNBC	538 (12.8)	122 (12.1)	138 (12.9)	146 (14.4)	132 (12.1)	
Unknown	73 (1.7)	19 (1.9)	14 (1.3)	12 (1.2)	28 (2.6)	

Lymph vascular Invasion						0.44
Yes	1,149 (27.4)	285 (28.2)	312 (29.1)	266 (26.3)	286 (26.2)	
No	3,018 (72.0)	723 (71.5)	755 (70.3)	738 (73.0)	802 (73.4)	
Unknown	23 (0.6)	3 (0.3)	7 (0.7)	8 (0.8)	5 (0.5)	
Multiplicity						0.01
Yes	913 (21.8)	250 (24.7)	245 (22.8)	209 (20.7)	209 (19.1)	
No	3,274 (78.1)	760 (75.2)	829 (77.2)	803 (79.4)	882 (80.8)	
Unknown	3 (0.1)	1 (0.1)	0 (0.0)	0 (0.0)	1 (0.1)	
Nuclear grade						0.35
Low	847 (20.2)	229 (22.7)	220 (20.5)	187 (18.5)	211 (19.3)	
Intermediate	1,942 (46.4)	466 (46.1)	499 (46.5)	485 (47.9)	492 (45.0)	
high	1,393 (33.3)	315 (31.2)	353 (32.9)	337 (33.3)	388 (35.5)	
Unknown	8 (0.2)	1 (0.1)	2 (0.2)	3 (0.3)	2 (0.2)	

* Comorbidity was measured from 2009 year. †Hypertension, diabetes and vascular event

Total cholesterol (mg/dl): Q_1=170, Q_2=191, Q_3=215

LDL cholesterol (mg/dl): Q_1=98, Q_2=118, Q_3=139

HDL cholesterol (mg/dl): Q_1=49, Q_2=59, Q_3=69

Triglyceride (mg/dl): Q_1=63, Q_2=87, Q_3=123

TG/HDL: Q_1=0.97, Q_2=1.47, Q_3=2.33

Non HDL cholesterol: Q_1=109, Q_2=132, Q_3=156

BCS: breast-conserving surgery; ER: estrogen receptor; IDC: invasive ductal carcinoma; ILC: invasive lobular carcinoma; LVI: lymphovascular invasion; NG: nuclear grade; PR: progesterone receptor; RM: resection margin; TM: total mastectomy; TNBC: triple negative breast cancer

Supplementary Table 2. Characteristics by quartile of LDL at baseline in breast cancer patients who underwent a surgery.

	**Quartile I (*n* = 1,030) n (%)**	**Quartile II (*n* = 1,054) n (%)**	**Quartile III (*n* = 1,050) n (%)**	**Quartile IV (*n* = 1,056) n (%)**	***p*-Value**
Age, Mean (SD)	48.8 (10.8)	50.6 (9.5)	52.8 (9.0)	54.5 (8.8)	<0.001
BMI, Mean (SD)	22.8 (3.1)	23.2 (3.0)	23.8 (3.2)	24.6 (3.1)	<0.001
Menopausal status					<0.001
Yes	398 (38.6)	488 (46.3)	609 (58.0)	722 (68.4)	
No	630 (61.2)	564 (53.5)	439 (41.8)	329 (31.2)	
Unknown	2 (0.2)	2 (0.2)	2 (0.2)	5 (0.5)	
Comorbidity (*n* = 2,946)*					0.37
Yes†	155 (21.1)	152 (19.9)	179 (23.5)	150 (21.9)	
No	580 (78.9)	613 (80.1)	583 (76.5)	534 (78.1)	
Surgery type (*n* = 4,188)					0.29
Mastectomy	304 (29.6)	272 (25.8)	292 (27.8)	298 (28.2)	
Lumpectomy	724 (70.4)	782 (74.2)	758 (72.2)	758 (71.8)	
Stage					0.22
1	525 (51.0)	521 (49.4)	509 (48.5)	495 (46.9)	
2	396 (38.5)	416 (39.5)	440 (41.9)	427 (40.4)	
3	109 (10.6)	117 (11.1)	101 (9.6)	134 (12.7)	
ER					<0.001
Positive	820 (79.6)	802 (76.1)	771 (73.4)	762 (72.2)	
Negative	208 (20.2)	252 (23.9)	278 (26.5)	294 (27.8)	
Unknown	2 (0.2)	0 (0.0)	1 (0.1)	0 (0.0)	
PR					<0.001
Positive	773 (75.1)	744 (70.6)	701 (66.8)	676 (64.0)	
Negative	255 (24.8)	310 (29.4)	348 (33.1)	380 (36.0)	
Unknown	2 (0.2)	0 (0.0)	1 (0.1)	0 (0.0)	
HER2					<0.001
Positive	163 (15.8)	220 (20.9)	223 (21.2)	236 (22.35)	
Negative	844 (81.9)	823 (78.1)	814 (77.5)	794 (75.19)	
Unknown	23 (2.2)	11 (1.0)	13 (1.2)	26 (2.46)	
Subtype					<0.001
Luminal A	726 (70.5)	691 (65.6)	669 (63.7)	651 (61.7)	
Luminal B	87 (8.5)	113 (10.7)	104 (9.9)	102 (9.7)	
HER2 type	76 (7.4)	107 (10.2)	119 (11.3)	134 (12.7)	
TNBC	118 (11.5)	132 (12.5)	145 (13.8)	143 (13.5)	
Unknown	23 (2.2)	11 (1.0)	13 (1.2)	26 (2.5)	
Lymph vascular Invasion					0.11

Yes	289 (28.1)	310 (29.4)	275 (26.2)	275 (26.0)	
No	739 (71.8)	737 (69.9)	765 (72.9)	777 (73.6)	
Unknown	2 (0.2)	7 (0.7)	10 (1.0)	4 (0.4)	
Multiplicity					0.03
Yes	255 (24.8)	236 (22.4)	212 (20.2)	210 (19.9)	
No	774 (75.2)	818 (77.6)	838 (79.8)	844 (80.0)	
Unknown	1 (0.1)	0 (0.0)	0 (0.0)	1 (0.1)	
Nuclear grade					0.01
Low	247 (24.0)	215 (20.4)	194 (18.5)	191 (18.1)	
Intermediate	481 (46.7)	478 (45.4)	501 (47.7)	482 (45.6)	
high	300 (29.1)	360 (34.2)	351 (33.4)	382 (36.2)	
Unknown	2 (0.2)	1 (0.1)	4 (0.4)	1 (0.1)	

* Comorbidity was measured from 2009 year. †Hypertension, diabetes and vascular event

Total cholesterol (mg/dl): Q_1=170, Q_2=191, Q_3=215

LDL cholesterol (mg/dl): Q_1=98, Q_2=118, Q_3=139

HDL cholesterol (mg/dl): Q_1=49, Q_2=59, Q_3=69

Triglyceride (mg/dl): Q_1=63, Q_2=87, Q_3=123

TG/HDL: Q_1=0.97, Q_2=1.47, Q_3=2.33

Non HDL cholesterol: Q_1=109, Q_2=132, Q_3=156

Supplementary Table 3. Characteristics by quartile of HDL at baseline in breast cancer patients who underwent a surgery.

	**Quartile I (*n* = 949) n (%)**	**Quartile II (*n* = 1,144) n (%)**	**Quartile III (*n* = 1,010) n (%)**	**Quartile IV (*n* = 1,087) n (%)**	***p*-Value**
Age, Mean (SD)	53.6 (10.1)	52.2 (10.0)	51.0 (9.4)	50.1 (9.3)	<0.001
BMI, Mean (SD)	24.8 (3.2)	24.0 (3.2)	23.2 (2.9)	22.5 (2.8)	<0.001
Menopausal status					<0.001
Yes	566 (59.6)	624 (54.6)	507 (50.2)	520 (47.8)	
No	379 (39.9)	518 (45.3)	500 (49.5)	565 (52.0)	
Unknown	4 (0.4)	2 (0.2)	3 (0.3)	2 (0.2)	
Comorbidity (*n* = 2,946)*					<0.001
Yes†	185 (29.7)	194 (23.8)	141 (19.4)	116 (14.8)	
No	437 (70.3)	622 (76.2)	585 (80.6)	666 (85.2)	
Surgery type (*n* = 4,188)					0.14
Mastectomy	283 (29.8)	291 (25.5)	279 (27.6)	313 (28.8)	
Lumpectomy	666 (70.2)	851 (74.5)	731 (72.4)	774 (71.2)	
Stage					<0.001
1	416 (43.8)	528 (46.2)	523 (51.8)	583 (53.6)	
2	408 (43.0)	469 (41.0)	389 (38.5)	413 (38.0)	
3	125 (13.2)	147 (12.9)	98 (9.7)	91 (8.4)	
ER					0.03
Positive	696 (73.3)	835 (73.0)	784 (77.6)	840 (77.2)	
Negative	253 (26.7)	308 (26.9)	225 (22.3)	246 (22.6)	
Unknown	0 (0.0)	1 (0.1)	1 (0.1)	1 (0.1)	
PR					0.09
Positive	650 (68.5)	757 (66.2)	717 (71.0)	770 (70.8)	
Negative	299 (31.5)	386 (33.7)	292 (28.9)	316 (29.1)	
Unknown	0 (0.0)	1 (0.1)	1 (0.1)	1 (0.1)	
HER2					0.49
Positive	181 (19.1)	249 (21.8)	187 (18.5)	225 (20.7)	
Negative	753 (79.4)	878 (76.8)	803 (79.5)	841 (77.4)	
Unknown	15 (1.6)	17 (1.5)	20 (2.0)	21 (1.9)	
Subtype					0.10
Luminal A	607 (64.0)	719 (62.9)	686 (67.9)	725 (66.7)	
Luminal B	86 (9.1)	118 (10.3)	90 (8.9)	112 (10.3)	
HER2 type	95 (10.0)	131 (11.5)	97 (9.6)	113 (10.4)	
TNBC	146 (15.4)	159 (13.9)	117 (11.6)	116 (10.7)	
Unknown	15 (1.6)	17 (1.5)	20 (2.0)	21 (2.0)	

Lymph vascular Invasion					0.28
Yes	269 (28.4)	329 (28.8)	259 (25.6)	292 (26.9)	
No	678 (71.4)	808 (70.6)	742 (73.5)	790 (72.7)	
Unknown	2 (0.2)	7 (0.6)	9 (0.9)	5 (0.5)	
Multiplicity					0.80
Yes	205 (21.6)	241 (21.1)	232 (23.0)	235 (21.6)	
No	744 (78.4)	902 (78.9)	777 (76.9)	851 (78.4)	
Unknown	0(0.0)	1 (0.1)	1 (0.1)	1 (0.1)	
Nuclear grade					<0.001
Low	160 (16.9)	213 (18.6)	221 (21.9)	253 (23.3)	
Intermediate	439 (46.3)	532 (46.5)	487 (48.2)	484 (44.5)	
high	347 (36.6)	398 (34.8)	300 (29.7)	348 (32.0)	
Unknown	3 (0.3)	1 (0.1)	2 (0.2)	2 (0.2)	

* Comorbidity was measured from 2009 year. †Hypertension, diabetes and vascular event

Total cholesterol (mg/dl): Q_1=170, Q_2=191, Q_3=215

LDL cholesterol (mg/dl): Q_1=98, Q_2=118, Q_3=139

HDL cholesterol (mg/dl): Q_1=49, Q_2=59, Q_3=69

Triglyceride (mg/dl): Q_1=63, Q_2=87, Q_3=123

TG/HDL: Q_1=0.97, Q_2=1.47, Q_3=2.33

Non HDL cholesterol: Q_1=109, Q_2=132, Q_3=156

Supplementary Table 4. Characteristics by quartile of triglyceride at baseline in breast cancer patients who underwent a surgery.

	**Quartile I (*n* = 1,029) n (%)**	**Quartile II (*n* = 1,053) n (%)**	**Quartile III (*n* = 1,048) n (%)**	**Quartile IV (*n* = 1,060) n (%)**	***p*-Value**
Age, Mean (SD)	46.9 (8.8)	50.6 (9.0)	53.4 (9.5)	55.7 (9.5)	<0.001
BMI, Mean (SD)	22.1 (2.6)	23.2 (2.9)	23.9 (3.1)	25.0 (3.3)	<0.001
Menopausal status					<0.001
Yes	329 (32.0)	507 (48.2)	627 (59.8)	754 (71.1)	
No	699 (67.9)	540 (51.3)	417 (39.8)	306 (28.9)	
Unknown					
Comorbidity (*n* = 2,946)*					<0.001
Yes†	76 (10.0)	132 (17.5)	181 (24.7)	247 (35.4)	
No	685 (90.0)	622 (82.5)	552 (75.3)	451 (64.6)	
Surgery type (*n* = 4,188)					0.55
Mastectomy	286 (27.8)	278 (26.4)	306 (29.2)	296 (27.9)	
Lumpectomy	742 (72.2)	775 (73.6)	741 (70.8)	764 (72.1)	
Stage					0.01
1	553 (53.7)	505 (48.0)	509 (48.6)	483 (45.6)	
2	379 (36.8)	439 (41.7)	416 (39.7)	445 (42.0)	
3	97 (9.4)	109 (10.4)	123 (11.7)	132 (12.5)	
ER					<0.001
Positive	814 (79.1)	810 (76.9)	763 (72.8)	768 (72.5)	
Negative	213 (20.7)	243 (23.1)	284 (27.1)	292 (27.6)	
Unknown	2 (0.2)	0 (0.0)	1 (0.1)	0 (0.0)	
PR					<0.001
Positive	768 (74.6)	739 (70.2)	698 (66.6)	689 (65.0)	
Negative	259 (25.2)	314 (29.8)	349 (33.3)	371 (35.0)	
Unknown	2 (0.2)	0 (0.0)	1 (0.1)	0 (0.0)	
HER2					0.02
Positive	176 (17.1)	209 (19.9)	226 (21.6)	231 (21.8)	
Negative	827 (80.4)	828 (78.6)	810 (77.3)	810 (76.4)	
Unknown	26 (2.5)	16 (1.5)	12 (1.2)	19 (1.8)	
Subtype					<0.001
Luminal A	706 (68.6)	702 (66.7)	669 (63.8)	660 (62.3)	
Luminal B	101 (9.8)	102 (9.7)	100 (9.5)	103 (9.7)	
HER2 type	75 (7.3)	107 (10.2)	126 (12.0)	128 (12.1)	
TNBC	121 (11.8)	126 (12.0)	141 (13.5)	150 (14.2)	
Unknown	26 (2.5)	16 (1.5)	12 (1.2)	19 (1.8)	
Lymph vascular Invasion					0.45

Yes	305 (29.6)	289 (27.5)	281 (26.8)	274 (25.9)	
No	718 (69.8)	756 (71.8)	762 (72.7)	782 (73.8)	
Unknown	6 (0.6)	8 (0.8)	5 (0.5)	4 (0.4)	
Multiplicity					0.01
Yes	251 (24.4)	237 (22.5)	229 (21.9)	196 (18.5)	
No	777 (75.5)	816 (77.5)	819 (78.2)	862 (81.4)	
Unknown	1 (0.1)	0 (0.0)	0 (0.0)	1 (0.1)	
Nuclear grade					0.08
Low	225 (21.9)	229 (21.8)	192 (18.3)	201 (19.0)	
Intermediate	479 (46.6)	497 (47.2)	469 (44.8)	497 (46.9)	
high	323 (31.4)	327 (31.1)	384 (36.6)	359 (33.9)	
Unknown	2 (0.2)	0 (0.0)	3 (0.3)	3 (0.3)	

* Comorbidity was measured from 2009 year. †Hypertension, diabetes and vascular event

Total cholesterol (mg/dl): Q_1=170, Q_2=191, Q_3=215

LDL cholesterol (mg/dl): Q_1=98, Q_2=118, Q_3=139

HDL cholesterol (mg/dl): Q_1=49, Q_2=59, Q_3=69

Triglyceride (mg/dl): Q_1=63, Q_2=87, Q_3=123

TG/HDL: Q_1=0.97, Q_2=1.47, Q_3=2.33

Non HDL cholesterol: Q_1=109, Q_2=132, Q_3=156
